# Association analysis of melanophilin (*MLPH*) gene expression and polymorphism with plumage color in quail

**DOI:** 10.5194/aab-66-131-2023

**Published:** 2023-03-22

**Authors:** Zhiwen Yuan, Xiaohui Zhang, Youzhi Pang, Yanxia Qi, Qiankun Wang, Yunqi Hu, Yiwei Zhao, Shiwei Ren, Linke Huo

**Affiliations:** 1 College of Animal Science and Technology, Henan University of Science and Technology, Luoyang 471003, China; 2 Luoyang Key Laboratory of Animal Genetics and Breeding, Luoyang 471003, China

## Abstract

We explore the relationship between the melanophilin (*MLPH*) gene and quail plumage color and provide a reference for subsequent quail plumage color breeding. In this experiment, real-time quantitative PCR (RT-qPCR) technology was used to analyze the relative mRNA expression levels of Korean quail (maroon) and Beijing white quail embryos at different developmental stages. Two single-nucleotide polymorphisms (SNPs) in the *MLPH* gene were screened based on the RNA-sequencing (RNA-Seq) data of skin tissues of Korean quail and Beijing white quail during the embryonic stage. Kompetitive allele-specific PCR (KASP) technology was used for genotyping in the resource population, and correlation analysis was carried out with the plumage color traits of quail. Finally, bioinformatics was used to predict the effects of these two SNPs on the structure and function of the encoded protein. The results showed that the expression level of the *MLPH* gene during embryonic development of Beijing white quail was significantly higher than that of Korean quail ( P<0.01 ). The frequency distribution of the three genotypes (CC, CA and AA) of the Beijing white quail at the c.1807C > A mutation site was significantly different from that of the Korean quail ( P<0.01 ). The frequency distribution of the three genotypes (GG, GA and AA) of the Beijing white quail at the c.2129G > A mutation site was significantly different from that of the Korean quail ( P<0.01 ). And there was a significant correlation between the c.1807C > A mutation site and the white plumage phenotype. Bioinformatics showed that SNP1 (c.1807C > A) was a neutral mutation and that SNP2 (c.2129G > A) was a deleterious mutation. The prediction of protein conservation showed that the mutation sites of coding proteins R603S and G710D caused by SNP1 (c.1807C > A) and SNP2 (c.2129G > A) were highly conserved.

## 1 Introduction

Plumage color is an important appearance and quality trait of poultry, which can be used not only for variety identification but also for molecular genetic breeding markers (Mastrangelo et al., 2020). In poultry genetic breeding, plumage color has always been an important breeding indicator, which is closely related to consumer preferences and has important economic value (Xi et al., 2020). Plumage formation is closely related to melanin deposition and degradation. Avian plumage color differences are mainly due to the uneven distribution of melanin in the body. Melanin is divided into eumelanin and pheomelanin. As the main component of avian plumage color formation, melanin deposition and degradation will have an important impact on the formation of plumage color (Nam et al., 2021; Li et al., 2012). Melanin synthesis is mainly by amino acid synthesis of tyrosine in the body, through the tyrosinase catalytic body tyrosine hydroxylation and a series of biochemical reactions (Raghunath et al., 2015; Galeb et al., 2021). Once functional disorder occurs in these processes, it will lead to melanin synthesis defects, which will lead to abnormal pigmentation (Wakabayashi et al., 2021). Quail is an important avian animal, and the genetic mechanism of plumage color is very complex. Studies have shown that *TYR*, *TYRP1*, *MC1R* and *ASIP* genes are related to the formation of plumage color in quail (Li et al., 2019; Xu et al., 2013).

The melanophilin (*MLPH*) gene is a Rab effector protein involved in melanosome transport, which can regulate skin pigmentation during melanosome transport (Manakhov et al., 2019; Myung et al., 2021). As a protein complex involving myosin Va (*MyoVa*) and *Rab27a*, *MLPH* protein also enables the movement and migration of melanin bodies in melanocytes (Lee et al., 2019). Studies have shown that *MLPH* mutations affect the transport of melanin from the skin to the hair follicles, leading to the loss of melanin transport function (Zhang et al., 2020; Moravčíková et al., 2021; Doig et al., 2013). At present, studies have found that *MLPH* gene mutation will lead to coat color dilution in cats, dogs and rabbits (Ishida et al., 2006; Lehner et al., 2013; Bauer et al., 2018). However, the correlation between the *MLPH* gene mutation and avian plumage color is rare; the correlation between the *MLPH* gene mutation and quail plumage color is especially less reported. Studying the differences in the *MLPH* gene mutations in different quail populations can help us to further understand the genetic mechanism of quail plumage color.

## 2 Materials and methods

### 2.1 Animals

The study protocol was approved by the Animal Care Committee of Henan University of Science and Technology. The study was conducted in the animal genetic breeding laboratory of the College of Animal Science and Technology, Henan University of Science and Technology. The study was conducted from May to September 2022. Korean quail (the color of the plumage is maroon) and Beijing white quail (the color of the plumage is white) eggs (36 each) were purchased from Quail Seed Industry Co., Ltd., Henan University of Science and Technology. The quail eggs were incubated in an incubator at (37 ± 1) ${}^{\circ }$ C with relative humidity of 50 %–65 %. Meanwhile, 15 mg samples of embryonic skin tissue were taken at days 8, 10, 12 and 14 and then frozen in liquid nitrogen for subsequent RNA extraction. In total, 350 eggs of Korean quail and 350 eggs of Beijing white quail were purchased, and the skin tissues of each embryo were collected on the 10th day of incubation and frozen in liquid nitrogen for subsequent genomic DNA extraction.

### 2.2 Total RNA extraction and complementary DNA (cDNA) synthesis

The skin tissue of quail embryos was crushed and ground, and the total RNA was extracted by TRIzol reagent (Invitrogen, Thermo Fisher Scientific, USA) according to the manufacturer's instructions. The RNA concentration was detected by a NanoDrop D2000 (Thermo Fisher Scientific, USA), and the RNA quality was detected by 1 % agarose gel electrophoresis. Total RNA was reverse-transcribed into first-strand cDNA using the “PrimeScript™ IV 1st strand cDNA Synthesis Mix” reverse transcription kit (TaKaRa, Japan) and stored at - 20 ${}^{\circ }$ C.

### 2.3 Detection of *MLPH* mRNA expression by RT-qPCR

Using *EIF2S3* (Yuan et al., 2022) as the reference gene, real-time quantitative PCR (RT-qPCR) was used to detect the expression level of the *MLPH* gene in different developmental stages of Korean quail and Beijing white quail embryos. The *MLPH* and *EIF2S3* primers were designed by Primer 5.0 software and synthesized by Wuhan Aoke Dingsheng Biotechnology Co., Ltd. (Aoke Dingsheng, Wuhan, China). The primer information is shown in Table 1. The RT-qPCR reaction system (20 µ L) consisted of 1 µ L of cDNA template, 10 µ L of SYBR^®^ Premix Ex Taq™ II (2 × ), 0.7 µ L of forward primers, 0.7 µ L of reverse primers and 7.6 µ L of ddH ${}_{2}$ O (double-distilled). The RT-qPCR reaction conditions were as follows: pre-denaturation at 95 ${}^{\circ }$ C for 3 min, denaturation at 95 ${}^{\circ }$ C for 20 s, annealing for 30 s (the annealing temperature of each gene is shown in Table 1) and extension at 72 ${}^{\circ }$ C for 20 s (40 cycles); fluorescence signals were collected during the extension phase. The melting curve was increased by 0.5 ${}^{\circ }$ C every 5 s from 65 to 95 ${}^{\circ }$ C.

**Table 1:** The information of KASP and qPCR primers.

Gene	Primer sequences (5 ${}^{\prime }$ –3 ${}^{\prime }$ )	Length (bp)	Annealing temperature ( ${}^{\circ }$ C)	Purpose
*MLPH*	F: AGGTGGTTCGGCGTGACTTC	245	64	qPCR
	R: GCCCTGCTCCCTCTTGTTG			
*EIF2S3*	F: GATTGACCCAACTTTGTGCC	147	60	qPCR
	R: TTCTTGTCTCCTTCAGTGCG			
*MLPH* (c.1807C > A)	F: AGGAGAGGAGTGAATAGAAAG	233	56.3	KASP
	R1: TCAGCCCTTACCTCACG			
	R2: TCAGCCCTTACCTCACT			
*MLPH* (c.2129G > A)	F: AAATGTTGTAGGTTTGGGTGGT	243	57.3	KASP
	R1: GCGGTCAAAAGAATCATCAAAAC			
	R2: GCGGTCAAAAGAATCATCAAAAT			

### 2.4 Genomic DNA extraction and detection

The embryonic skin tissues of Korean quail and Beijing white quail were crushed and ground, and the genomic DNA was extracted by an animal genomic DNA extraction kit (TaKaRa, Japan). DNA concentration was detected by a NanoDrop D2000, and DNA quality was detected by 1 % agarose gel electrophoresis.

### 2.5 *MLPH* gene polymorphism detection

Two single-nucleotide polymorphisms (SNPs) in the *MLPH* gene were screened based on the RNA-sequencing (RNA-Seq) data of skin tissues of Korean quail and Beijing white quail during the embryonic stage. The gene sequence information of Japanese quail was obtained from the National Center for Biotechnology Information (NCBI) database. Two pairs of primers (one positive and two negative) were designed using Primer 5.0 software, and the primers were synthesized by Wuhan Aoke Dingsheng Biotechnology Co., Ltd. (Table 1). The *MLPH* genes of Korean quail and Beijing white quail were genotyped by Kompetitive allele-specific PCR (KASP) technology. The KASP reaction system conditions were as follows: KASP 2 × Master mix V3 (5 µ L), forward universal primer F (0.9 µ L), reverse typing primer R1 (0.3 µ L), reverse typing primer R2 (0.3 µ L), template DNA (1 µ L) and ddH ${}_{2}$ O (2.5 µ L); the total volume was 10 µ L. The reaction conditions were as follows: the first step was 94 ${}^{\circ }$ C for 6 min; the second step was 94 ${}^{\circ }$ C denaturation for 20 s, 61 ${}^{\circ }$ C annealing for 60 s (from the second cycle, 0.6 ${}^{\circ }$ C per cycle), 10 cycles; the third step was 94 ${}^{\circ }$ C denaturation for 20 s, 55 ${}^{\circ }$ C annealing for 60 s, 26 cycles; and step four was 37 ${}^{\circ }$ C for 5 min (KASP typing data should generally be read below 40 ${}^{\circ }$ C). The amplified products were read by a fluorescence quantitative PCR instrument (Bio-Rad, USA) to obtain the relative fluorescence unit (RFU) values of each sample. Genotyping was generated using R, version 4.1.2, ggplot2 package.

### 2.6 Bioinformatics of the *MLPH* gene encoding protein

The protein sequence of the *MLPH* gene was analyzed by bioinformatics. The software packages and their website information are shown in Table 2.

**Table 2:** *MLPH* gene sequence bioinformatics software packages and their websites.

Software name	Software website (last access: 13 August 2022)
Signal5.0	http://www.cbs.dtu.dk/services/SignalP
TMHMM	http://www.cbs.dtu.dk/services/TMHMM
PROVEAN	http://provean.jcvi.org/seq_submit.php
PolyPhen 2.0	http://genetics.bwh.harvard.edu/pph2/
SNAP2	https://www.rostlab.org/services/snap/
I-Mutant	http://gpcr2.biocomp.unibo.it/cgi/predictors/I-Mutant3.0/I-Mutant3.0.cgi
MutPred2	http://mutpred.mutdb.org/index.html
ConSurf	https://consurf.tau.ac.il/2016/

### 2.7 Statistics and analyses

The 2 ${}^{-\Delta \Delta \mathrm{Ct}}$ method was used to calculate the relative expression of the gene (Livak et al., 2001). The t test was performed by SPSS 20.0 software, and a graph was drawn by GraphPad Prism 8.0. A chi-squared test was performed by SPSS 20.0 software to evaluate the allele frequency of each SNP and its association with plumage color. Polymorphic information content (PIC) was calculated using PICcalc software (Nagy et al., 2012).

## 3 Results

### 3.1 mRNA expression level of the *MLPH* gene

**Figure 1:**
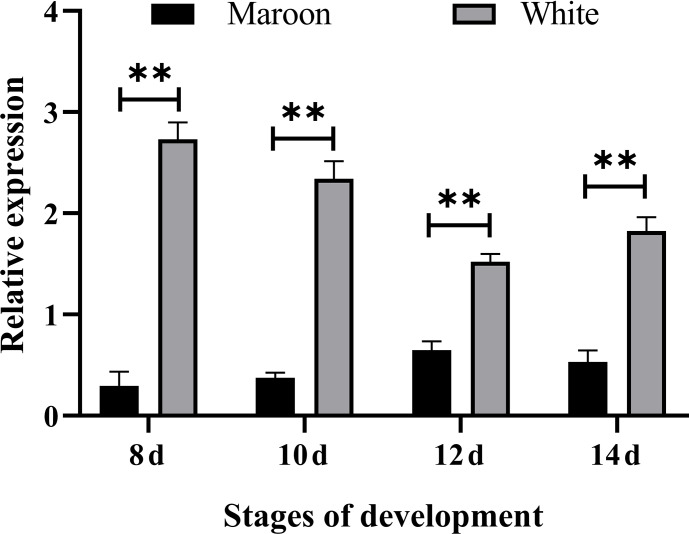
The mRNA expression levels of the *MLPH* gene in different embryonic development stages of Korean quail and Beijing white quail. Symbols ${}^{**}$ mean that the difference between quail embryos with different plumage colors at the same developmental stage is significant ( P<0.01 ).

RT-qPCR results showed that the expression levels of the *MLPH* gene in Korean quail and Beijing white quail at different stages of embryonic development were significantly different, and the relative expression level of Beijing white quail was significantly higher than that of Korean quail (Fig. 1). This indicates that the *MLPH* gene inhibits melanin synthesis in quail plumage color formation.

### 3.2 Genotype analysis and genetic information analysis of the *MLPH* gene mutation sites

**Figure 2:**
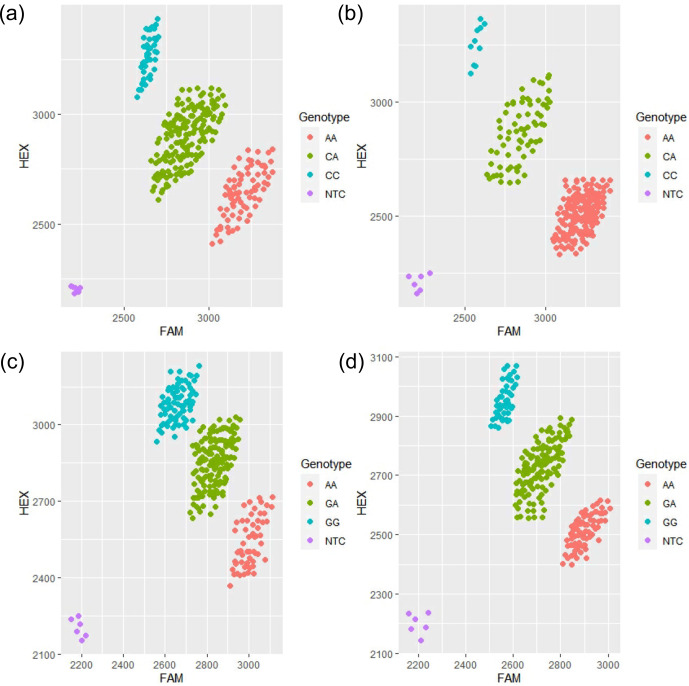
Principal component analysis (PCA) typing diagram of each mutation site of the *MLPH* gene. **(a)** PCA graph of c.1807C > A for Korean quail; **(b)** PCA graph of c.1807C > A for Beijing white quail; **(c)** PCA graph of c.2129G > A for Korean quail; **(d)** PCA graph of c.2129G > A for Beijing white quail.

The results showed that there were three genotypes (CC, CA and AA) in exon 13 and three genotypes (GG, GA and AA) in exon 16 of the *MLPH* gene (Fig. 2). According to the genetic information of the *MLPH* gene mutation site, the dominant allele of the c.1807C > A mutation site in the quail population was A, and the dominant allele frequency was 0.69. The dominant allele of the mutation site c.2129G > A was A, and the dominant allele frequency was 0.51. By calculating the polymorphism information content of Korean quail and Beijing white quail, the two mutation sites of the *MLPH* gene were moderate polymorphisms (0.25 < PIC < 0.5). A Hardy–Weinberg equilibrium test on each mutation site showed that the quail population was in Hardy–Weinberg equilibrium at both mutation sites ( P>0.05 ), as shown in Table 3.

**Table 3:** Analysis of different genotype populations at *MLPH* gene mutation sites.

Loci	Genotypic frequencies			Allele frequencies		PIC	Hardy–Weinberg	
							equilibrium	
							$\chi^{2}$	P
c.1807C > A	0.095(CC)	0.435(CA)	0.470(AA)	0.31(C)	0.69(A)	0.34	0.084	0.772
c.2129G > A	0.223(GG)	0.537(GA)	0.240(AA)	0.49(G)	0.51(A)	0.37	3.097	0.078

$\chi^{2}$ : chi-squared test; P : probability.

### 3.3 Association analysis of the *MLPH* gene polymorphism with plumage color traits

The association analysis between the *MLPH* gene and quail plumage color traits showed that there was a significant correlation between the *MLPH* gene c.1807C > A mutation site and quail plumage color traits ( P<0.01 ), where allele A in the Beijing white quail population occupies an absolute advantage, indicating that the mutation site will hinder the pigment deposition of quail plumage. The frequency distribution of the three genotypes of c.2129G > A in Beijing white quail was significantly different from that in Korean quail ( P<0.01 ). Allele G had an advantage in Korean quail, and allele A had an advantage in Beijing white quail, indicating that the plumage color traits of maroon plumage and white plumage at this mutation site were correlated with the frequencies of alleles G and A, respectively (Table 4).

**Figure 3:**
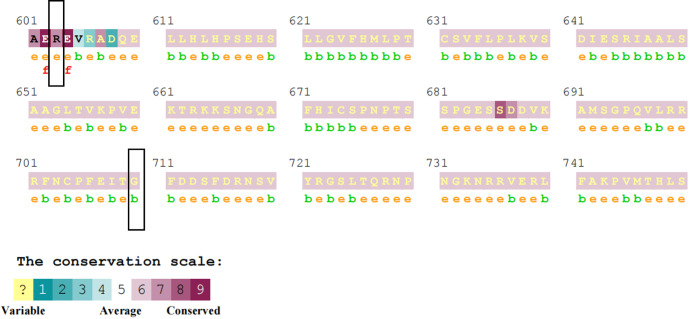
Conservation analysis of the *MLPH* protein. Different scores output by ConSurf represent different degrees of conservation. The lower the score, the lower the conservation; the higher the score, the higher the conservation. The black boxes represent the two mutation sites of R603S and G710D.

### 3.4 Bioinformatics of the *MLPH* protein

The analysis showed that the *MLPH* protein did not have the signal peptide and belonged to non-secretory proteins. The prediction results of TMHMM online software showed that the *MLPH* protein had no transmembrane region, and the total probability of the *MLPH* protein being located on the membrane cytoplasm side was 0.00078.

Three nonsynonymous SNP (nsSNP) online functional prediction software packages and one stability prediction software package were used to analyze the two nsSNP stability prediction software packages, and the results were slightly different. Comprehensive analysis showed that SNP1 (c.1807C > A) was a neutral mutation and that SNP2 (c.2129G > A) was a deleterious mutation (Table 5). The prediction results of MutPred online software showed that the R603S and G710D sites may not cause changes in membrane protein structure (Table 6).

The prediction results of ConSurf online software showed that the R603S site score is 7, which belongs to the conservative site. The G710D site score is 6, which belongs to the neutral site (Fig. 3).

**Table 4:** Correlation analysis of quail *MLPH* gene polymorphisms and plumage color traits.

Loci	Phenotype	Total	Genotypic frequencies		Allele frequencies				
		number							
								$\chi^{2}$	P
c.1807C > A	Maroon	287	0.146(42)(CC)	0.586(168)(CA)	0.268(77)(AA)	0.44(C)	0.56(A)	100.237	0.000
	White	260	0.039(10)(CC)	0.269(70)(CA)	0.692(180)(AA)	0.17(C)	0.83(A)		
c.2129G > A	Maroon	287	0.268(77)(GG)	0.537(154)(GA)	0.195(56)(AA)	0.54(G)	0.46(A)	10.509	0.005
	White	260	0.173(45)(GG)	0.538(140)(GA)	0.289(75)(AA)	0.44(G)	0.56(A)		

$\chi^{2}$ : chi-squared test; P : probability.

## 4 Discussion

In this experiment, RT-qPCR was used to analyze the relative expression levels of the *MLPH* gene in Korean quail and Beijing white quail at different developmental stages. The results showed that the relative expression level of the *MLPH* gene in Beijing white quail was significantly higher than that in Korean quail, which is consistent with the research results that the relative expression level of the *MLPH* gene in white goat skin tissue was significantly higher than that in black goat and brown goat skin tissues (Fu, 2013). Lee et al. (2015) also showed that the relative expression level of the *MLPH* gene in Korean zebra cattle was significantly higher than that in brown cattle. The relative expression level of the *MLPH* gene was the highest on the eighth day of embryonic development in Beijing white quail, indicating that the gene was active at this time and affected the transportation and synthesis of melanin, thereby affecting the plumage color phenotype of quails. It was speculated that the occurrence of the white plumage phenotype of quails might be related to the high expression of the gene. Studies have shown that *MLPH* gene expression was detected in skin tissue of Zhedong white goose (Liu et al., 2016). This is also consistent with the results of the high expression of the *MLPH* gene in the skin tissue of Beijing white quail. The above studies have shown that the *MLPH* gene is related to the light color phenotype in animals, but its main expression and regulation mechanism need further study.

**Table 5:** Prediction results of destructiveness of the *MLPH* gene nsSNPs.

SNP site	Amino acid variation	PROVEAN	PolyPhen 2.0	SNAP2	I-Mutant 3.0
SNP1 (c.1807C > A)	R603S	3.890	0.000	- 57	- 0.57
SNP2 (c.2129G > A)	G710D	- 1.832	0.002	22	- 1.23

PROVEAN: score ≤-2.5 is a deleterious mutation, and score >-2.5 is a neutral mutation. PolyPhen-2: score <0.50 is benign; score ≥ 0.05 is destructible. SNAP2: score > 0 is influential; score < 0 variation is neutral. I-Mutant 3.0: score <-0.5 means reducing protein stability; - 0.5 ≤ score ≤ 0.5 means variation is neutral; score > 0.5 means increasing protein stability.

**Table 6:** Effects of the *MLPH* gene deleterious nsSNPs on protein tertiary structure.

SNP site	Amino acid variation	MutPred2 score	Effect
SNP1 (c.1807C > A)	R603S	0.055	No
SNP2 (c.2129G > A)	G710D	0.230	No

Some studies have found that the mutation of the *MLPH* gene is related to the fading of animal coat color or plumage color. Posbergh et al. (2020) found that a mutation site (g.3451931C > A) of the *MLPH* gene in Jacob sheep led to the premature introduction of terminator codons into the protein, resulting in the loss of its function, thus causing the black coat of Jacob sheep to fade. The polymorphism analysis of the *MLPH* gene in rabbits with different coat colors showed that the mutation site of the *MLPH* gene (g.232766A < T) was related to the white phenotype of rabbits (Jia et al., 2021). Studies have shown that *MLPH* gene mutation can lead to melanosome transport defects, and the mutation site C.1909A > G was detected to cause plumage color fading in Anyi tile-like gray chicken, resulting in the “five-gray” phenotype (Xu et al., 2016). Vaez et al. (2008) determined that the mutation site of the *MLPH* gene (C.C103T) could lead to amino acid changes (R35W) by comparative analysis of chicken candidate gene sequences, and there was a significant correlation between the mutation site and the chicken plumage color dilution phenotype. Wang et al. (2022) found that the mutation of the *MLPH* gene was closely related to the lavender dilution phenotype of Baicheng You chicken. The results of this experiment also showed that the c.1807C > A mutation site of the quail *MLPH* gene was significantly correlated with its white plumage phenotype. The mutation sites of the *MLPH* gene found in the above study are not consistent with those in this study, which may be due to the different mutation sites of the *MLPH* gene between different species. The above studies also showed that *MLPH* gene mutation can cause animal coat color or plumage color fading. In addition to the *MLPH* gene, there are some other genes associated with avian plumage color. Noor et al. (2021) found that *PMEL17* gene mutation was associated with the plumage color of Bangladeshi indigenous chicken, and the plumage color of indigenous chicken was associated with production performance. The *MLPH* gene was also studied in Japanese quail. Minvielle et al. (2009) found that a single mutation of the *MLPH* gene could lead to dilution of plumage color in Japanese quail. Bed'hom et al. (2012) also found that the complex mutation of the *MLPH* gene was related to the dilution of Japanese quail plumage color, which is consistent with the results of this experiment.

The frequency distributions of the three genotypes in Korean quail at two mutation sites (c.1807C > A and c.2129G > A) of the *MLPH* gene in this study were significantly different from those in Beijing white quail ( P<0.01 ). Bioinformatic prediction showed that c.1807C > A was a neutral mutation and that c.2129G > A was a deleterious mutation. Conservative prediction of protein showed that the mutation sites of encoding proteins R603S and G710D caused by the two sites were a conservative site and neutral site, respectively, indicating that the two mutation sites might not cause changes in protein structure. However, association analysis showed that allele A at the c.1807C > A mutation site had an absolute advantage in the white quail population, suggesting that the occurrence of the white phenotype in the quail population might be related to the fact that allele A at this mutation site was a dominant gene in the population. Combined with the above studies, it can be inferred that *MLPH* gene mutation may play an important regulatory role in quail plumage color fading.

## 5 Conclusions

The relative expression level of the *MLPH* gene in Beijing white quail was significantly higher than that in Korean quail. The c.1807C > A mutation site in the quail *MLPH* gene was significantly correlated with its white plumage phenotype, and the c.2129G > A mutation site was significantly associated with its plumage color phenotype. SNP1 (c.1807C > A) was a neutral mutation, and SNP2 (c.2129G > A) was a deleterious mutation. The mutation sites of coding proteins R603S and G710D caused by SNP1 (c.1807C > A) and SNP2 (c.2129G > A) were highly conserved. The results can provide a reference for subsequent animal plumage color or coat color breeding.

## Data Availability

RNA-Seq data are from the NCBI database (accession number: PRJNA756792). The remaining data are available from the corresponding author upon request.

## Code availability

The bioinformatics software packages used in this study can be used according to the URL provided in Table 2.
